# Impaired T helper cell responses in human immunodeficiency virus‐exposed uninfected newborns

**DOI:** 10.1002/iid3.507

**Published:** 2021-08-19

**Authors:** Yesenia Brito‐Pérez, Rodrigo T. Camacho‐Pacheco, Noemi Plazola‐Camacho, Diana Soriano‐Becerril, Irma A. Coronado‐Zarco, Gabriela Arreola‐Ramírez, Gabriela González‐Pérez, Alma Herrera‐Salazar, Julio Flores‐González, Mextli Y. Bermejo‐Haro, Brenda G. Casorla‐Cervantes, Ismael A. Soto‐López, Jessica Hernández‐Pineda, Claudia Sandoval‐Montes, Sandra Rodríguez‐Martínez, Ricardo Figueroa‐Damian, Ismael Mancilla‐Herrera

**Affiliations:** ^1^ Infectology and Immunology Department National Institute of Perinatology (INPer) Mexico City Mexico; ^2^ Departamento de Inmunología, Escuela Nacional de Ciencias Biológicas Instituto Politécnico Nacional Ciudad de México México; ^3^ Posgrado en Inmunología, Departamento de Inmunología, Escuela Nacional de Ciencias Biológicas Instituto Politécnico Nacional Mexico City Mexico; ^4^ Neonatology Department National Institute of Perinatology (INPer) Mexico City Mexico; ^5^ Department of Physiology and Cellular Development National Institute of Perinatology (INPer) Mexico City Mexico

**Keywords:** HIV‐exposed uninfected newborns, Th cells, Th_1_/Th_2_/Th_17_/Treg/CD4^+^CD25^++^ T cells and Th_1_ differentiation

## Abstract

**Introduction:**

HIV‐exposed uninfected (HEU) newborns suffer from higher risks of opportunistic infections during the first months of life compared to HIV‐unexposed uninfected (HUU) newborns. Alterations in thymic mass, amounts of T helper (Th) cells, T‐cell receptor diversity, and activation markers have been found in HEU newborns, suggesting alterations in T cell ontogeny and differentiation. However, little is known about the ability of these cells to produce specialized Th responses from CD4^+^ T cells.

**Method:**

To characterize the Th cell profile, we evaluated the frequency of Th_1_ (CD183^+^CD194^−^CD196^−^/CXCR3^+^CCR4^−^CCR6^−^), Th_2_ (CD183^−^CD194^+^CD196^−^/CXCR3^−^CCR4^+^CCR6^−^), Th_17_ (CD183^−^CD194^+^CD196^+^/CXCR3^−^CCR4^+^CCR6^+^), and CD4^+^CD25^++^ blood T‐cell phenotypes in 50 HEU and 25 HUU newborns. Early activation markers on CD4^+^ T cells and the Th cytokine profile produced from mononuclear cells under polyclonal T cell stimulation were also studied. Additionally, we probed the ability of CD4^+^ T cells to differentiate into interferon (IFN)‐γ‐producing Th_1_ CD4^+^ T cells *in vitro*.

**Results:**

Lower percentages of differentiated Th_1_, Th_2_, Th_17,_ and CD4^+^CD25^++^ T cells were found in blood from HEU newborns than in blood from HUU newborns. However, polyclonally stimulated Th cells showed a similar ability to express CD69 and CD279 but produced less secreted interleukin (IL)‐2 and IL‐4. Interestingly, under Th_1_ differentiation conditions, the percentages of CD4^+^IFN‐γ^+^ T cells and soluble IFN‐γ were higher in HEU newborns than in HUU newborns.

**Conclusion:**

HEU neonates are born with reduced proportions of differentiated Th_1_/Th_2_/Th_17_ and CD4^+^CD25^++^ T cells, but the intrinsic abilities of CD4^+^ T cells to acquire a Th_1_ profile are not affected by the adverse maternal milieu during development.

## Graphical abstract

The frequency of differentiated peripheral Th cells in HIV‐exposed uninfected (HEU) newborns is reduced compared to control newborns. The consequences of this poorly differentiated pool of Th cells in HEU newborns could contribute to the high susceptibility to infections during the first months of life

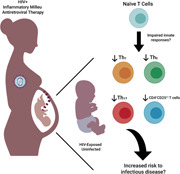

## 1 INTRODUCTION

One of the most concerning issues about human immunodeficiency virus (HIV) is the possibility of vertical transmission. Currently, the implementation of perinatal care, antiretroviral therapy, and cesarean delivery as well as restriction of breastfeeding have decreased the risk of transferring maternal viral infection to children.^1^ Despite no evidence of HIV infection, infants born to HIV‐1‐infected mothers (HIV‐exposed uninfected [HEU]) have demonstrated a greater risk of suffering infectious diseases, tripling the morbidity and mortality indexes at the first months of life compared to HIV‐unexposed uninfected (HUU) children.^2^, ^3^, ^4^

The relatively higher rates of infections in HEU children are associated with several altered components of the immune system, mainly abnormalities of the cellular adaptive responses that have been traced back to T cell ontogeny. Notably, the thymuses of HIV‐exposed infants have been reported to be smaller than those of HIV‐unexposed infants. Additionally, HEU neonates show the reduced thymic output of mature naïve T cells and reduced T‐cell receptor (TCR) diversity on naïve T cells.^5^, ^6^, ^7^ Additionally, compared to HUU infants, polyclonal cell stimulation in HEU infants causes increases in interferon (IFN)‐γ, interleukin (IL)‐2, and tumor necrosis factor (TNF)‐α, as well as higher T lymphoproliferative responses^8^, ^9^, ^10^, ^11^ and overexpression of activation markers, such as CD69, CD279, HLA‐DR, and CD38.^5^, ^12^ Despite these strong nonspecific responses, reduced cell‐mediated immunity to vaccines in HEU infants has been reported. After antigen‐specific stimulation, peripheral blood mononuclear cells (MCs) from HEU infants vaccinated with Bacillus Calmette‐Guérin and tetanus show a reduced frequency of proliferating T cells and a lower frequency of Th cells expressing effector cytokines.^13^, ^14^, ^15^, ^16^ Although these observations may appear to be contradictory, they all suggest that HIV maternal infection might negatively impact the development of optimal cellular immune responses to the antigenic challenge after birth.

On the basis of the profile of cytokine secretion, tropism, and type of promoted immune response, several Th cell responses have been described, including Th_1_/Th_2_/Th_17_ and regulatory T‐cell (Treg) responses. The appropriate development of this immunity is crucial to mount a specialized defense against pathogens and, in turn, to tolerate innocuous environmental and autologous antigens.^17^, ^18^, ^19^ The specialization of Th cells in healthy adults depends on the microenvironment surrounding naïve CD4^+^ T cells in mature lymphoid tissue, but the neonatal architecture of lymphoid tissues is in development and antigenically little experienced. Contrary to the idea that the prenatal condition is immunologically immature, the presence of fetal memory T cells before the second half of gestation has been reported.^20^, ^21^ At delivery, humans show preferential regulatory profile (Th_2_ and Treg) responses that gradually change to a Th_1_ response by the first week, and the equilibrium among all profiles is modeled during the first years of life.^22^, ^23^ However, chronic maternal viral infections, such as hepatitis B virus (HBV), can predispose naïve T cells to polarize toward a Th_1_ response early by disturbing the development of immunity to future antigenic challenges.^24^ Altogether, these findings suggest that the modeling of Th responses starts with gestation and maternal environment conditions as early immune responses of offspring can potentially promote healthy or immunologic disorders.

Chronic HIV maternal infection negatively impacts the cellular adaptive immunity of HEU infants as reflected by several alterations in the T‐cell subpopulations, but the characterization of specialized Th responses in this susceptible group has not been completely performed until now. In this study, we present the characterization of the naturally occurring Th profile of HEU newborns and the predisposition of CD4^+^ T cells from peripheral blood to acquire a Th_1_ phenotype *in vitro*.

## 2 MATERIAL AND METHODS

The present study was performed in accordance with the ethical standards in the Declaration of Helsinki and approved by the Research, Biosecurity, and Ethics Committees at the National Institute of Perinatology (INPer), Mexico City. The protocol was funded by projects 2019‐1‐31 and 2017‐2‐85 from the same institute.

### 2.1 Neonate selection criteria and blood samples

Fifty HIV‐1‐exposed neonates born to HIV‐infected mothers admitted at the INPer were included in the present work. The inclusion criteria were as follows: Neonates with term birth (≥37 weeks), singletons, normal birth weight, and no congenital abnormalities. Complications associated with maternal comorbidities were excluded, and all mothers received antiretroviral treatment during pregnancy. Upon admission to the INPer, all mothers were tested for HBV and hepatitis C virus (HCV) by serological assays. After the HEU's mothers signed informed consent forms, 500 μl of peripheral blood was obtained from neonates on the first day of birth. Afterward, all HEU infants were monitored bimonthly up to 2 years of age with clinical CD4 count and viral load tests to discard vertical HIV transmission. Twenty‐five umbilical cord blood samples from cesarian‐normal delivery HUUs were used as controls.

### 2.2 Th cell phenotyping

Twenty‐five microliters of peripheral blood from HEU newborns or umbilical cord blood from HUU newborns were incubated for 15 min at room temperature in the dark with titrated volumes of fluorochrome‐conjugated monoclonal antibodies according to the following panel: ‐CD3‐AF488 (Clone HIT3A, Cat. 300320; Biolegend), ‐CD4‐APC‐Cy7 (Clone SK3, Cat. 344616; Biolegend), ‐CD45RA‐PE‐CF594 (Clone HI100, Cat. 562298; BD Biosciences), ‐CD25‐PE (Clone MA251, Cat. 555432; BD Biosciences), ‐CD183(CXCR3)‐BV510 (Clone G025H7, Cat. 353726; Biolegend), ‐CD194(CCR4)‐APC (Clone L291H4, Cat. 359408; Biolegend), and –CD196(CCR6)‐PerCP‐Cy5.5 (Clone G034E3, Cat. 353406; Biolegend). After incubation, samples were fixed with 250 µl of 1× FACS lysing solution (Cat. 349202; BD Biosciences) and washed with 500 μl of FACSFlow solution (Cat. 342003; BD Biosciences). All samples were acquired and analyzed using a FACS ARIA III flow cytometer with DIVA V8.0.2 software (BD Biosciences). Appropriate compensation controls were used.

The Th profile was characterized using the next algorithm: Singlet cells were gated from a 45° diagonal line by the FSC‐A versus FSC‐H dot plot. The lymphoid region was gated as a homogeneous population with low size (FSC) and complexity (SSC). Memory Th cells were then determined to be double‐positive for CD3 and CD4 but negative for CD45RA. From memory Th cells, we identified CD4^+^CD25^++^ T cells as a representation of Treg cells.^25^, ^26^ From the CD4^+^CD25^−^ T cells, the Th_1_, Th_2_, and Th_17_ cells were identified by the CD183^+^CD194^−^CD196^−^ (CXCR3^+^CCR4^−^CCR6^−^), CD183^−^CD194^+^CD196^−^ (CXCR3^−^CCR4^+^CCR6^−^), CD183^−^CD194^+^CD196^+^(CXCR3^−^CCR4^+^CCR6^+^) phenotypes, respectively. In addition, Th_0_ cells were identified by the CD45RA^+^CD183^−^CD194^−^CD196^−^ (CD45RA^+^CXCR3^−^CCR4^−^CCR6^−^) phenotype (Figure S1a).

**Figure 1:**
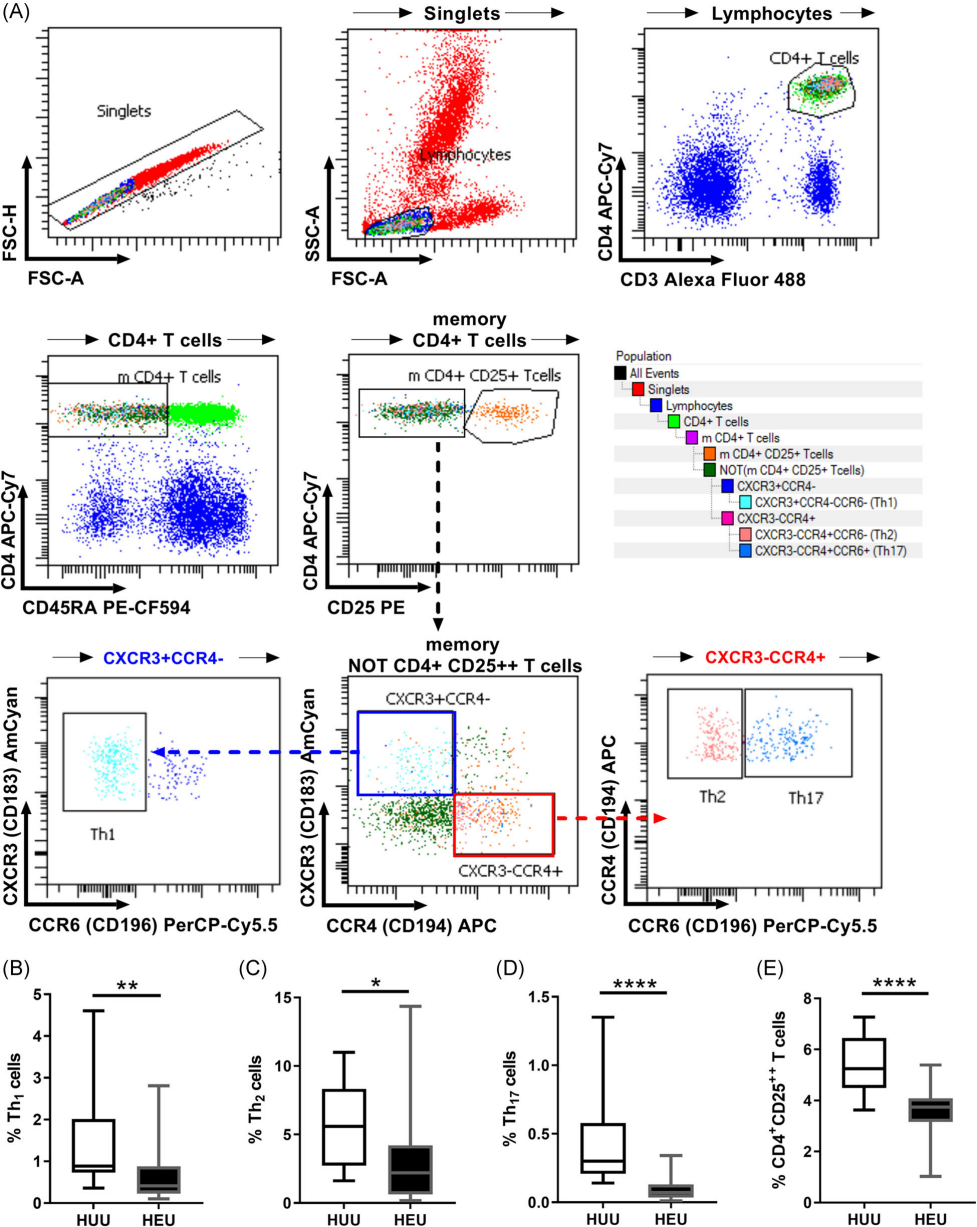
Low percentages of differentiated Th cells in HEU newborns. Peripheral blood from HEU or umbilical cord blood from HUU newborns was stained with monoclonal antibodies and analyzed by flow cytometry. (A) Representative gating strategy. From singlet memory, CD4^+^ T lymphocytes, subsets of Th cells were identified with the following phenotypes: Th_1_ (CD183^+^CD194^−^CD196^−^/CXCR3^+^CCR4^−^CCR6^−^); Th_2_ (CD183^−^CD194^+^CD196^−^/CXCR3^−^CCR4^+^CCR6^−^); Th_17_ (CD183^−^CD194^+^CD196^+^/CXCR3^−^CCR4^+^CCR6^+^); and CD4^+^CD25^++^ T cells. The percentages of (B) Th_1_, (C) Th_2,_ (D) Th_17_, and (E) CD4^+^CD25^++^ T cells among total CD4^+^ T lymphocytes from HEU and HUU newborns are shown. Error bars indicate the median and interquartile range. HEU, HIV‐exposed uninfected; HUU, HIV‐unexposed uninfected. *p* Values were calculated using the unpaired Mann–Whitney test. *p < 0.05, **p < 0.01, and ****p < 0.0001

### 2.3 Mononuclear cell separation and stimulation

Under sterile conditions, peripheral or umbilical cord blood samples from HEU or HUU neonates, respectively, were diluted (1:1) with sterile physiological saline solution (SPSS). MC isolation was then performed by a density gradient with Lymphoprep® solution (Cat. 07851; Stemcell Technologies) and centrifugation at 600*g* for 15 min at room temperature. The MC fraction was collected, washed with 500 µl of SPSS, and centrifuged at 400*g* for 5 min. Cellular viability and counting of resuspended cells were determined by trypan blue (0.4%) (Life Technologies) exclusion assay and analyzed in an automatized cell counter (Countess Invitrogen). After counting, 5 × 10^5^ MCs were polyclonally stimulated and incubated in 250 µl of Roswell Park Memorial Institute (RPMI) medium and 5 µl of ImmunoCult™ Human CD3/CD28/CD2 T‐Cell Activator (Cat. 10990; Stemcell Technologies) for 24 h at 37°C in a 5% CO_2_ environment. A separate culture was incubated without stimulus as a negative control. After incubation, cultures were centrifuged at 400*g* for 5 min. Supernatants were collected and stored at –20°C for cytokine quantification. The cellular fraction was superficially immunostained as described above using the following panel: anti‐CD3‐PE‐ef610 (Clone UCHT1, Cat. 4324117; eBioscience), ‐CD4‐APC‐Cy7 (Clone SK3, Cat. 344616; eBioscience), ‐CD19‐FITC (Clone SJ25C1, Cat. 340409; eBioscience), ‐CD16‐FITC (Clone SE, Cat. IM0814U; Beckman Coulter), ‐CD56‐BV421 (Clone HCD56, Cat. 318328; Biolegend), ‐CD69‐PERCPef710 (Clone FN50, Cat. 46‐0699‐42; eBioscience™), ‐CD279‐PE (Clone EH12.1, Cat. 560795; BD Biosciences), ‐CD45‐PECy7 (Clone HI30, Cat. 304016; Biolegend) and fixable and viability dye (FVD)/eFluor506 (Cat. 65‐08‐66‐14; eBiosciences). After immunostaining, samples were analyzed by flow cytometry.

For activated cell characterization, CD4^+^ T cells were determined from the live lymphoid region (gated as FVD‐negative events) by the CD45^Hi^CD19^−^CD16^−^CD56^−^CD3^+^CD4^+^ phenotype. The percentages of CD4^+^ T cells expressing activation markers (CD69 and CD279) were determined considering other live lymphocytes (CD19^+^ or CD16^+^ events) as references. CD69^+^ and CD279^+^ cells were then determined in CD4^+^ T cells (Figure S2).

### 2.4 Th_1_ cell differentiation

Five hundred thousand live MCs were incubated in 250 µl of RPMI medium supplemented with Th_1_ medium containing recombinant IL‐12, IL‐18, and anti‐IL‐4 antibodies (ImmunoCult™ Human Th_1_ Differentiation Supplement) and 5 µl of ImmunoCult™ Human CD3/CD28/CD2 T‐Cell Activator (Cat. 10990; Stemcell Technologies) for 7 days. In parallel, the same number of MCs was cultivated only in RPMI as a control. The medium was changed on the fourth day, and 6 h before the end of incubation, 1 µg/ml of the protein transport inhibitor, GolgiStop™ (Cat. 554724; BD Biosciences), was added. After incubation, cultures were centrifuged, and supernatants were stored for cytokine quantification. The cellular fraction was washed with 250 µl of Cell Staining Buffer (Cat. 420201; Biolegend), permeabilized with 250 µl of BD Cytofix/Cytoperm™ buffer (Cat. 554715; BD Biosciences), and incubated for 20 min at 4°C. Cells were then washed and incubated in the dark for 30 min at 4°C with 5 µl of monoclonal antibody mix containing the following antibodies: anti‐CD3‐PEef610 (Clone UCHT1, Cat. 4324117; eBioscience), ‐CD45‐PECy7 (Clone HI30, Cat. 304016; Biolegend), ‐CD4‐PerCP, ‐IFN‐γ‐FITC, Th1/Th2/Th17 phenotyping kit Cat. 560751; BD Biosciences) and FVD. After incubation, cells were washed with 500 μl of FACSFlow solution and analyzed by flow cytometry. The analysis was performed by selecting singlet cells and then live FVD‐negative lymphocytes and CD45^High^ events. Finally, Th cells were gated as double positives for CD3 and CD4, and the percentages of CD4^+^IFN‐γ^+^ T cells were determined.

### 2.5 Cytokine quantification in cell supernatants

Stored supernatants were thawed, and the concentrations of Th cytokines were determined using the Human CBA Th1/Th2/Th17 Kit (Cat. 560484; BD Biosciences). Briefly, 6.25 µl of supernatant was incubated in the dark with 3 ml of anti‐IL‐2, ‐IL‐4, ‐IL‐6, ‐IL‐10, ‐TNF‐α, ‐IFN‐γ, and ‐IL‐17a capture bead mix. Subsequently, 6.25 µl of detection antibody was added to the samples and incubated for 3 h at room temperature. After incubation, the beads were washed with wash buffer and centrifuged at 200*g* for 5 min. All samples were analyzed using a FACS ARIA III cytometer with DIVA V8.0.2 software (BD Biosciences). Log‐transformed data were used to obtain standard curves fitted to 10 discrete points using a four‐parameter logistic model. Concentrations were calculated using interpolation of the corresponding standard reference curves.

### 2.6 Statistical analyses

Differences between groups were calculated by the Mann–Whitney unpaired test, and differences between conditions in the same group were obtained by Wilcoxon paired test. Measures of central tendency are expressed as medians with interquartile ranges or means with standard errors and 95% confidence intervals. Significance for all tests was defined as *p* < 0.05. All analyses were performed using Prism software version 7 (GraphPad).

## 3 RESULTS

### 3.1 Characteristics of HEU and HUU newborns

It is well known that HEU newborns have higher rates of morbidity and mortality from recurrent infections associated with alterations in the immune system compared to HUU newborns. In the present study, we report the results from 25 HUU and 50 HEU newborns. Detailed descriptions of the mothers and neonates are summarized in Table 1. HIV‐infected mothers were younger than the controls (*p* < 0.05), but the number of prior pregnancies was comparable between groups. As expected, the percentage of CD4^+^ T cells from HEU's mothers was lower than that from control mothers (*p* < 0.0001). In addition, most HIV‐infected mothers had low viral loads (<200 copies/ml, 70%), and all mothers received a schedule of combined antiretrovirals. None of them was seropositive to HBV or HCV. Neonatal characteristics, such as gestational age and cephalic perimeter, were slightly lower in the HEU group (*p* < 0.05 for both) compared to the HUU group, whereas weight and length at birth were similar between both groups. Additionally, the CD4^+^ T‐cell counts in HEU newborns were in normal ranges, and the percentages of total Th cells and the frequency of memory CD4^+^ T cells were comparable between both newborn groups. During medical follow‐up throughout the first 2 years of life, none of the HEU infants showed signs of HIV infection.

**Table 1:** Demographic characteristics of mothers and HIV‐unexposed uninfected or HIV‐exposed uninfected newborns

	HUU (*n* = 25)	HEU (*n* = 50)	*p* Value^a^
Maternal characteristics			
Maternal age (years, mean ± SD)	31.4 (5.3)	26.7 (6.1)	0.0028**
Number of prior pregnancies (mean±SD)	2.8 (1.1)	2.4 (1.2)	0.2599
CD4 count (cells/mm^3^) (mean ± SD)	‐	1061 (377.8)	‐
Percentages of Th cells^b^ (mean±SD)	45.46 (6.4)	29.4 (13.8)	<0.0001****
Viral load (copies/ml), *n* (%)	‐	<200: 35 (70)	‐
		200–1000: 2 (4)	
		>1000: 9 (18)	
		Not sample: 4 (8)	
Antiretrovirals, *n* (%)	‐	Integrase INH: 21(42)	‐
		Protease INH: 18 (36)	
		Retrotranscriptase INH: 9 (18)	
		Unknown: 2 (4)	
Newborn characteristics			
Gestational age (weeks) (mean ± SD)	38.6 (1.1)	37.7 (1.8)	0.0248*
Birth weight (g) (mean ± SD)	2979 (374.9)	2815 (420.1)	0.0619
Cephalic perimeter (cm) (mean ± SD)	34.3 (1.3)	33.6 (1.5)	0.0423*
Length at birth (cm) (mean ± SD)	48.4 (2.2)	48.2 (2.2)	0.5160
CD4 count (cells/mm^3^) (mean ± SD)	‐	2214 (943.3)	‐
Percentages of Th cells^b^ (mean ± SD)	66.01 (8.7)	65.9 (11.8)	0.9429
Percentages of memory CD4^+^ T cells (CD45RA‐) (mean ± SD)	14.35 (5.5)	11.35 (7.1)	0.0966

Abbreviations: HEU, HIV‐exposed uninfected newborn; HUU, HIV‐unexposed uninfected newborn; INH, inhibitor; *n*, number; SD, standard deviation.

^a^ All p values were calculated by the Mann–Whitney test.

^b^ Percentages of Th cells among total lymphocytes from peripheral blood.

^*^ *p* < 0.05

^**^ *p* < 0.01

^****^ *p* < 0.0001.

### 3.2 HEU newborns have a low proportion of differentiated Th cells

Subsets of Th differentiated cells specialize in responding to pathogens. To determine the specialization degree of CD4^+^ T cells in HEU newborns, we performed a flow cytometry assay based on cell surface markers. Figure 1A shows the complete analysis for identifying Th cells in blood. Despite the low proportion of differentiated lymphocytes in neonatal blood (less than 15%), these cells were easily identified. By using this strategy, we observed that the frequencies of Th_1_ (0.62% vs. 1.4%; *p* = 0.0061; Figure 1B), Th_2_ (3.122% vs. 5.536%, *p* = 0.0126; Figure 1C), Th_17_ (0.097 vs. 0.4825, *p* = 0.0001; Figure 1D), and CD4^+^CD25^++^ T cells (3.617 vs. 5.475, *p* < 0.0001; Figure 1E) populations were lower in HEU newborns than in HUU newborns (Figure 1). Notably, Th_2_ and CD4^+^CD25^++^ T cells were enriched among Th cells in both groups.

### 3.3 Maternal HIV infection reduces the ability of MCs to produce Th cytokines in HEU newborns

**Figure 2:**
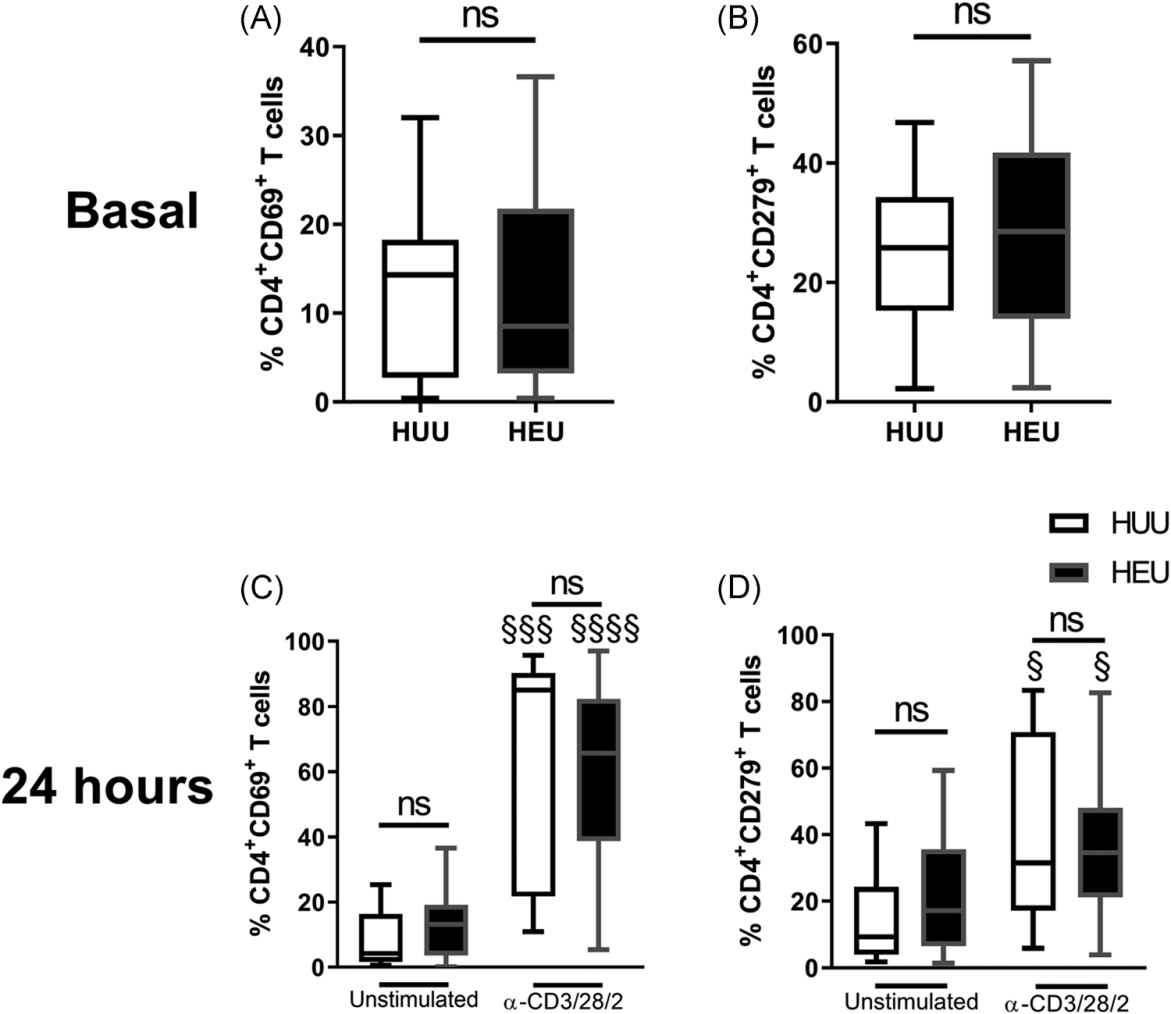
HEU newborns have similar percentages of Th cells expressing activation markers as HUU newborns. Th cells from blood mononuclear cells (MCs) of HEU and HUU newborns were immunostained for activation markers. The basal percentages of (A) CD69^+^ and (B) CD279^+^ Th cells are shown. Cells were incubated with or without anti‐CD3/CD28/CD2 for 24 h. The percentages of (C) CD69^+^ and (D) CD279^+^ Th cells are presented from unstimulated (left) and stimulated (right) conditions. Error bars indicate the median and interquartile range. *p* Values were calculated using an unpaired Mann–Whitney test to compare groups (HEU vs. HUU newborns). HEU, HIV‐exposed uninfected newborn; HUU, HIV‐unexposed uninfected newborn; ns, not significant. *p* Values were calculated using paired Wilcoxon tests to compare conditions (unstimulated vs. stimulated). The symbol "^§^" was used to show differences between stimulation conditions. ^§^ *p* < 0.05, ^§§^ *p* < 0.01, and ^§§§§^ *p* < 0.0001

To further investigate the functionality of HEU CD4^+^ T cells, we polyclonally stimulated T cells in MCs with anti‐CD3/CD28/CD2 and evaluated the expression of early and late activation markers associated with activation of CD4^+^ T cells at baseline and 24 h, and we also evaluated the Th cytokine profile that was produced after stimulation. The complete analysis identifying the cell activation markers of Th cells is shown in Figure S2. The basal percentages of CD69^+^ (Figure 2A) and CD279^+^ Th cells (Figure 2B) were similar in HEU newborns and HUU newborns. Additionally, stimulation increased the percentages of CD69‐ and CD279‐expressing CD4^+^ T cells in both HUU and HEU newborns, but the same levels of activation were observed among them (Figures 2C,D). These data indicated that the activation ability of CD4^+^ T cells is not compromised by HIV maternal infection.

**Figure 3:**
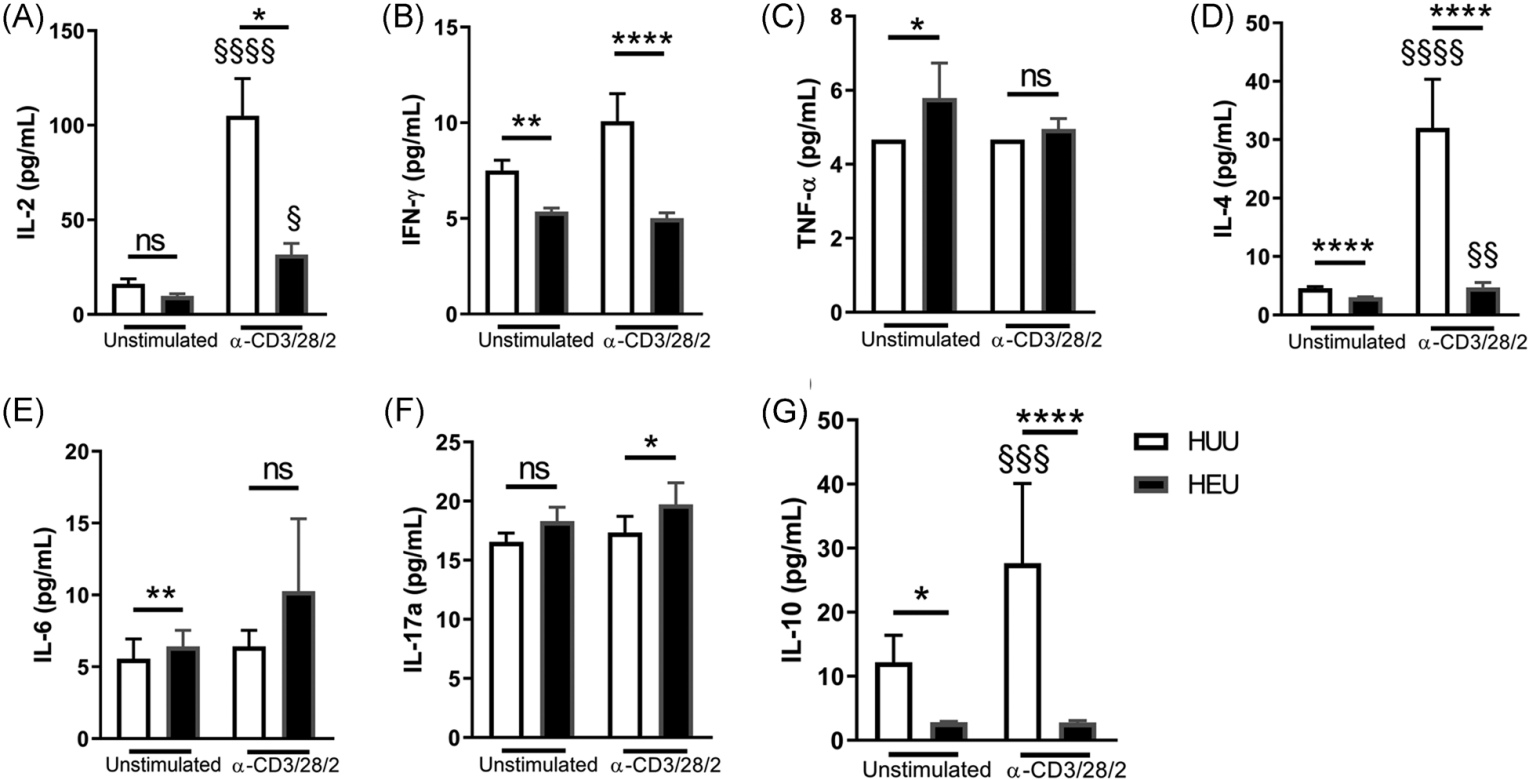
HEU newborns produce lower levels of cytokines than HUU newborns. After the mononuclear cell (MC) stimulation, supernatants were collected, and classical Th cytokine concentrations were quantified by multiplex immunoassays. The concentrations of (A) IL‐2, (B) IFN‐γ, (C) TNF‐α, (D) IL‐4, (E) IL‐6, (F) IL‐17a, and (G) IL‐10 from unstimulated (left) and stimulated (right) conditions are presented. Bars indicate the mean and standard error. *p* Values were calculated using unpaired Mann–Whitney test to compare groups (HEU vs. HUU newborns). HEU, HIV‐exposed uninfected newborn; HUU, HIV‐unexposed uninfected newborn; IFN‐γ, interferon‐γ; IL, interleukin; ns, not significant. *p < 0.05, **p < 0.01, and ****p < 0.0001. *p* Values were calculated using paired Wilcoxon tests to compare conditions (unstimulated vs. stimulated). ^§^ *p* < 0.05, ^§§^ *p* < 0.01,^§§§^ *p* < 0.001, and ^§§§§^ *p* *<* 0.0001

Twenty‐four hours after T cell stimulation, MC cultures from control newborns had higher concentrations of IL‐2 (Figure 3A), IL‐4 (Figure 3D), and IL‐10 (Figure 3G) but lower concentrations of IFN‐γ (Figure 3B), TNF‐α (Figure 3C), IL‐6 (Figure 3E), and IL‐17a (Figure 3F). In contrast, HEU newborn MCs showed a limited ability to produce IL‐2 or IL‐4 compared to that generated by the control group (Figures 3A,D). Moreover, the inducible response to produce IFN‐γ and IL‐10 was completely suppressed in HEU MCs, but at the same time, HEU MCs produced TNF‐α and IL‐6 without stimulation (Figures 3C,E).

In addition, to limit the possibility that cells other than Th cells produce cytokines, we normalized the concentration of each cytokine to the number of classical corresponding Th cells. Interestingly, higher levels of inflammatory cytokines (IFN‐γ and IL17a) were produced by differentiated cells in HEU newborns after stimulation, whereas regulatory cytokines (IL‐4 and IL‐10) showed the inverse behavior (Figure S3). Together, these results suggested that Th cells from HEUs are more inflammatory but that the pool of Th lymphocytes does not reach the number of HUU effector cells, possibly affecting the mechanisms of differentiation.

### 3.4 The intrinsic ability of CD4^+^ T cells to differentiate into the Th_1_ phenotype is not affected in HEU newborns

**Figure 4:**
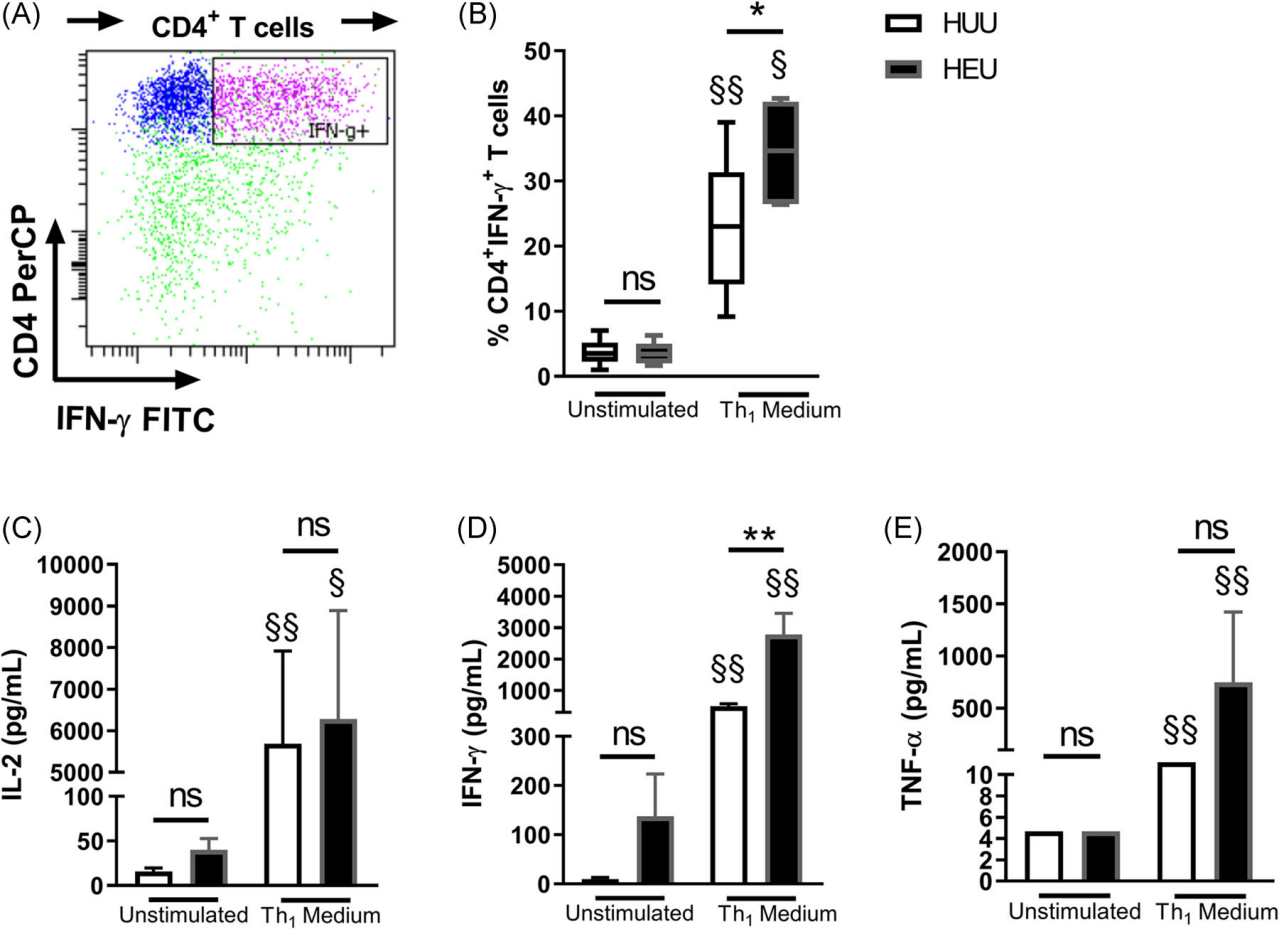
Th_1_‐differentiated MCs in HEU newborns have a higher capacity for IFN‐γ production. MCs were cultivated with or without differentiation Th_1_ medium for 7 days. The percentages of Th cells expressing IFN‐γ were then analyzed. (A) The representative gating analysis of cytokine‐producing CD4^+^ T cells and the percentages of (B) CD4^+^IFN‐γ^+^ T cells in HUU and HEU newborns are presented. Error bars indicate the median and interquartile range. The (C) IL‐2, (D) IFN‐γ, and (E) TNF‐α Th_1_ cytokines were quantified from culture supernatants. Bars indicate the mean and standard error. *p* Values were calculated using unpaired Mann–Whitney test to compare groups (HEU vs. HUU newborns). FITC, fluorescein isothiocyanate; HEU, HIV‐exposed uninfected newborn; HUU, HIV‐unexposed uninfected newborn; IFN‐γ, interferon‐γ; IL, interleukin; MC, mononuclear cell; ns, not significant; TNF‐α, tumor necrosis factor‐α. *p < 0.05, **p < 0.01. *p* Values were calculated using paired Wilcoxon tests to compare conditions (unstimulated vs. differentiated). ^§^ *p* < 0.05 and ^§§^ *p* < 0.01

The lower frequency of Th cells suggests that naïve CD4^+^ T cells from HEUs cannot specialize in Th phenotypes. Therefore, to explore the Th phenotypes, we evaluated the capacity of blood CD4^+^ T cells from HEUs to differentiate into the Th_1_ phenotype. Figure 4A shows that CD4^+^ T cells were the main producers of IFN‐γ among T cells. Contrary to the idea that the neonatal immune system is immature, we observed that CD4^+^ T cells from both HEU and HUU neonates were differentiated into IFN‐γ^+^‐producing cells (Figure 4B). Furthermore, T cells had the same ability to proliferate in both groups (Figure S4). Remarkably, the percentage of CD4^+^ T cells that produce IFN‐γ under Th_1_ polarization was higher in HEU newborns than in the control HUU group (34.48% vs. 23.54%; *p* = 0.0420).

Additionally, higher concentrations of soluble IL‐2 (Figure 4C), IFN‐γ (Figure 4D), and TNF‐α (Figure 4E) were observed in supernatants of differentiated MCs in both newborn groups. Correspondingly to the higher ability of CD4^+^ T cells to differentiate into IFN‐γ^+^‐producing cells, the concentration of IFN‐γ in supernatant from HEU newborn cultures was higher than that produced by HUU newborns (2780 vs. 499.4 pg/ml; *p* = 0.0070).

These results indicated that Th responses are affected by *in vivo* mechanisms of differentiation in HEU newborns. To support this hypothesis, we also observed that the frequency of blood Th_0_ cells (CD45RA^+^CXCR3^−^CCR4^−^CCR6^−^) was increased in HEU newborns, suggesting that T cells do not obtain sufficient *in vivo* stimulation to differentiate into Th effector cells (Figure S1b). Together, these results suggested that the inherent ability of HEU CD4^+^ T cells to specialize in the Th_1_ profile is not affected by maternal infection.

## 4 DISCUSSION

Recently, perinatal care has reduced the vertical transmission rates of HIV to less than 1%.^27^ However, the impact of the gestational environment in which the HEU newborn develops conditions of HIV that result in increased morbidity and mortality rates of infectious diseases remains unknown.^28^, ^29^, ^30^, ^31^ In several reports, this susceptibility has been associated with effects on the CD4^+^ T‐cell compartment. Our results showed a reduction in the frequency of peripheral Th_1_, Th_2_, Th_17,_ and CD4^+^CD25^++^ T cells but an increased ability of neonatal lymphocytes to differentiate into IFN‐γ‐producing CD4^+^ T cells. These contrasting results suggested that the intrinsic ability of T cells to differentiate in HEU newborns is not compromised by maternal infection but that the precursor mechanisms leading to *in vivo* differentiation may be notably affected by the adverse maternal milieu.

Previous studies have reported lower numbers of CD4^+^ T cells in HEU infants and newborns.^11^, ^32^, ^33^ These results have been explained by alterations in thymus development and functionality in HEU children.^5^, ^7^ In contrast, we observed that the frequencies of total CD4^+^ T cells were similar to those of control neonates. The differences may be associated with the provenance and maternal health status of the women studied herein. Although most reports address disadvantageous cities characterized by poor health care services and a high incidence of opportunistic infections, we included a group of patients who visited a higher health institution in a location with a remarkably low incidence of vertical transmission and maternal complications due to HIV infection.^34^ Additionally, the HEU mothers from this study were not significantly affected by the infection as they showed healthy counts of CD4^+^ T cells and most of them had low HIV viral loads.

In addition to the similar numbers of total CD4^+^ T cells, HEU newborns had similar frequencies of memory CD4^+^ T cells compared to HUU newborns. Although the presence of these cells at birth is not fully understood, it suggests that offspring are exposed to antigens since gestation. The presence of fetal memory T cells producing TNF‐α and IFN‐γ has been reported since the early second trimester of pregnancy.^20^, ^21^ Other studies have reported a high number of memory T cells in HEU infants, but similar to the case of low counts of total CD4^+^ T cells, their increases are correlated with the degree to which the mother is affected by HIV.^29^, ^35^

Among memory lymphocytes, we identified the Th_1_, Th_2_, Th_17,_ and CD4^+^CD25^++^ T phenotypes in blood, and we observed lower percentages of all these differentiated cells in HEU newborns. Interestingly, the phenotypes were mainly Th_2_ and CD4^+^CD25^++^ T cells in both groups. In peripheral blood, CD127^low^CD25^+^Foxp3^+^ Tregs are the most frequent lymphocytes among CD4^+^CD25^++^T cells.^26^ Therefore, we did not exclude the possibility that most CD4^+^CD25^++^ T cells evaluated in the HEU and HUU newborns were Treg cells, and we are currently investigating these cells in our laboratory. Previous reports have indicated that in the first week after birth, humans favor an immunoregulatory milieu mediated by Th_2_ and Treg responses, presumably due to the avoidance of strong inflammatory responses that may be induced when facing early antigenic challenges that could last throughout the first years of life.^21^, ^36^, ^37^ In this regard, it has been reported that Tregs are the most frequent cells colonizing peripheral tissues after birth (30%–40% among CD4^+^ T cells).^38^ In the case of HEU children who were gestated in an inflammatory environment with a placenta more permeable to maternal antigens,^39^, ^40^ we hypothesized that the frequency of Th cells, mostly Th_1_, is increased in HEU newborns due to increased antigenic exposure during gestation. Contrary to this idea, the percentages of all subtypes of Th cells were inferior in HEU newborns compared to control newborns. Therefore, we hypothesized that these results may be accompanied by alterations in the competence of activation and production of Th cytokines. Similar to previous reports,^5^, ^12^ we observed that the ability to express activation markers (CD69 or CD279) was not affected by the maternal condition of HIV infection. However, the ability of T cells to produce most of the Th cytokines was limited in HEU newborns, which may be explained by the low frequency of differentiated Th cells found in blood.

Reports of thymus from HEU fetuses and infants have shown important differences in size,^7^, ^33^ which may be due to an inflammatory gestational environment causing thymic disturbance and lymphocytic dysfunction.^41^, ^42^, ^43^, ^44^, ^45^ These observations may attribute to the lower frequency of differentiated Th cells in HEU newborns. Therefore, we evaluated the ability of CD4^+^ T cells to differentiate toward Th_1_ lymphocytes as an example of antiviral responses. Interestingly, CD4^+^ T cells from HEUs not only lost their ability to differentiate without affecting the competency to proliferate but also had increased frequencies of IFN‐γ‐producing CD4^+^ T cells. Preferential Th_1_ responses in neonates have been previously described due to maternal chronic HBV infections.^24^ We excluded the possibility that this result was due to HBV because all mothers included in our study were serologically negative for this virus. Therefore, we did not exclude the possibility that antiviral maternal responses during gestation predispose offspring to develop antiviral‐oriented responses. According to this hypothesis, a recent study has demonstrated that most T‐cell clonotypes from HEU newborns are enriched against viral antigens.^6^ The question of whether the CD4^+^IFN‐γ^+^ T cells induced in HEU cells correspond to enriched antiviral clonotypes remains unanswered and is currently being explored by our laboratory group.

Our results suggested that the intrinsic ability of CD4^+^ T cells to acquire a Th phenotype in HEU newborns is not limited by HIV maternal infection, but the lower frequency of differentiated cells may be due to *in vivo* effects that induce these specialized responses, mainly by innate immune responses. Previous studies have reported low percentages of NK cells with reduced capacities to produce IFN‐γ in HEU newborns.^46^ Additionally, IL‐12 production by cord blood mononuclear cells (CBMCs) is reduced in HEU newborns.^47^ Thus, abnormalities in IL‐12 regulation may contribute to the decreased cellular responses observed in HIV‐exposed infants. Some of these effects on innate cells may be attributed to antiretroviral drugs, which have been suggested as factors that compromise the response of the infant immune system and are partially responsible for poor outcomes.^32^ For example, zidovudine, a molecule that binds to leukocyte DNA, reduces the amount of platelets and neutrophils in HEU newborns and persists until 18 months of age.^48^, ^49^ Additionally, the combination of zidovudine and lamivudine provokes neutropenia, anemia, and neurologic complications related to mitochondrial dysfunction.^50^, ^51^ In our cohort of studies, the schedule of antiretroviral treatments was heterogeneous, but the results presented in this study were not correlated with specific treatment (data not shown). Although antiviral treatments can affect quantitative and qualitative innate responses, we cannot disregard the direct effect of maternal infection on innate immune responses or the altered development of lymphoid organs that should allow mounting an adequate immune response. However, these assumptions must be explored in depth.

To support the hypothesis that Th responses are affected by *in vivo* mechanisms of differentiation in HEU newborns, we compared the frequency of CD45RA^+^CXCR3^−^CCR4^−^CCR6^−^ T cells in both groups. These cells, recognized as Th_0_, are antigen‐primed T cells that receive a short stimulation from the TCR or cytokine milieu but do not acquire effector function and remain in a nonpolarized state.^52^ We observed that the percentages of Th_0_ cells were increased in HEU infants, suggesting that T cells do not receive sufficient stimulation to differentiate into Th effector cells.

Our study had several limitations. We used samples from different cell compartments from our study groups. Peripheral blood samples were used from HEU newborns to avoid the risk of contamination by maternal blood and HIV transmission, whereas we used umbilical cord blood samples from clinically healthy children because peripheral blood samples cannot be acquired due to bioethical factors. However, the comparison of different blood cell compartments, such as umbilical cord and peripheral blood T lymphocytes, is comparable during this short period of time after birth.^53^, ^54^ In addition, the cephalic perimeter was lower in HEU newborns than in the control group in our study cohort. These differences may be associated with less time of gestation. Even though all newborns were term, the neonatal characteristics of the HEU newborns were determined according to healthy Mexican and international child standards.^55^, ^56^ We exclusively explored the Th_1_ response to determine the antiviral response that could be triggered by gestational exposure to HIV as had previously been observed with HBV.^24^ We also explored the Th_2_ and Th_17_ responses in this group. Our results may help to elucidate the mechanisms in which maternal infections influence the immune responses of offspring against the first antigenic experiences and establish susceptibility to pathogen infections. Finally, to the best of our knowledge, this was the first time that this set of Th cells was described at the beginning of life using minimally invasive procedures, thereby allowing the study of neonatal pathologies of the adaptive immune response and the consequences of maternal diseases during pregnancy using our analytic strategy to characterize Th cells.

In conclusion, we demonstrated that the frequency of differentiated peripheral Th cells in HEU newborns is reduced compared to that in control newborns, resulting in a lower ability to produce effector cytokines. We also showed that this disadvantage is not caused by the intrinsic ability of CD4^+^ T cell differentiation. The consequences of this poorly differentiated pool of Th cells may contribute to the high susceptibility to infections during the first months of life.

## CONFLICT OF INTERESTS

The authors declare that there are no conflict of interests.

## AUTHOR CONTRIBUTIONS

Ismael Mancilla‐Herrera conceived and designed the study. Yesenia Brito‐Pérez and Rodrigo T. Camacho‐Pacheco performed the experiments and wrote the first version of the manuscript. Yesenia Brito‐Pérez, Rodrigo T. Camacho‐Pacheco, Julio Flores‐González, Mextli Y. Bermejo‐Haro, Brenda G. Casorla‐Cervantes, and Ismael A. Soto‐López analyzed and interpreted the data. Noemi Plazola‐Camacho, Irma A. Coronado‐Zarco, and Gabriela Arreola‐Ramírez contributed to the clinical evaluation of the patients. Diana Soriano‐Becerril, Gabriela González‐Pérez, Alma Herrera‐Salazar, Jessica Hernández‐Pineda, Claudia Sandoval‐Montes, Sandra Rodríguez‐Martínez, and Ricardo Figueroa‐Damian contributed to the critical revision of the report. All authors reviewed and approved the final version of the manuscript.

## Supporting information

Supplementary information.

Supplementary information.

Supplementary information.

Supplementary information.

Supplementary information.

## Data Availability

The data that support the findings of this study are available from the corresponding author upon reasonable request.
