# Supplementary figures and images for: Impaired T helper cell responses in human immunodeficiency virus‐exposed uninfected newborns
Source: Immun Inflamm Dis. 2021 Aug 19;9(4):1541–53. doi: 10.1002/iid3.507 (PMC8589403; doi:10.1002/iid3.507)

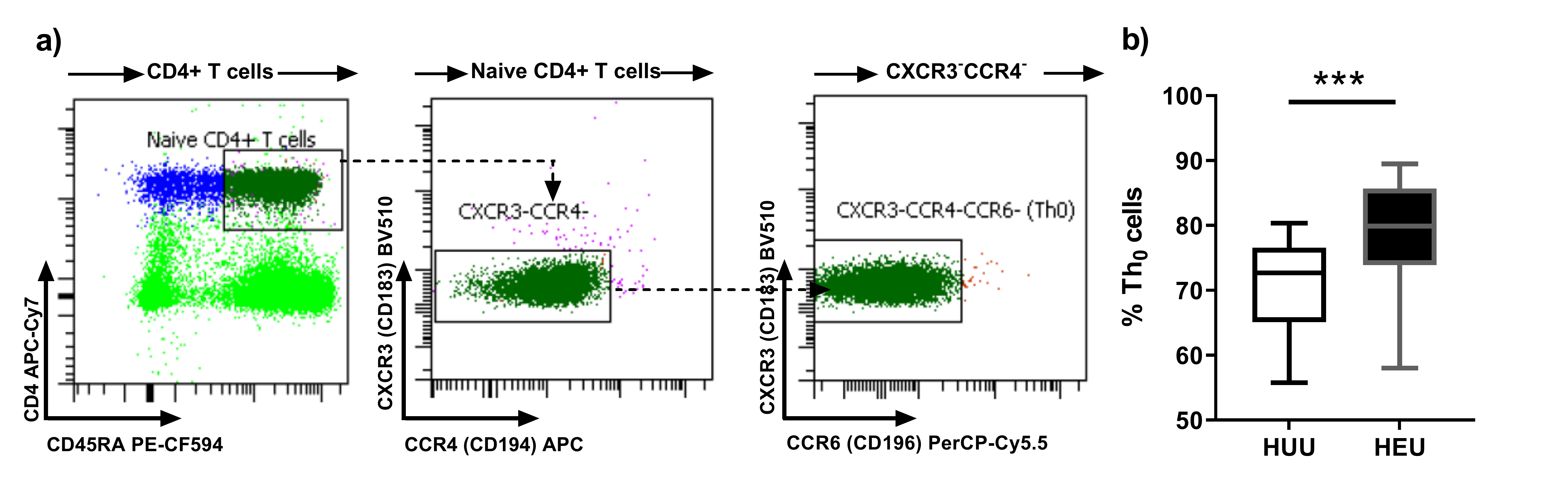

Supplement: Supplementary file 1 — Supplementary information. [file IID3-9-1541-s003.tif]

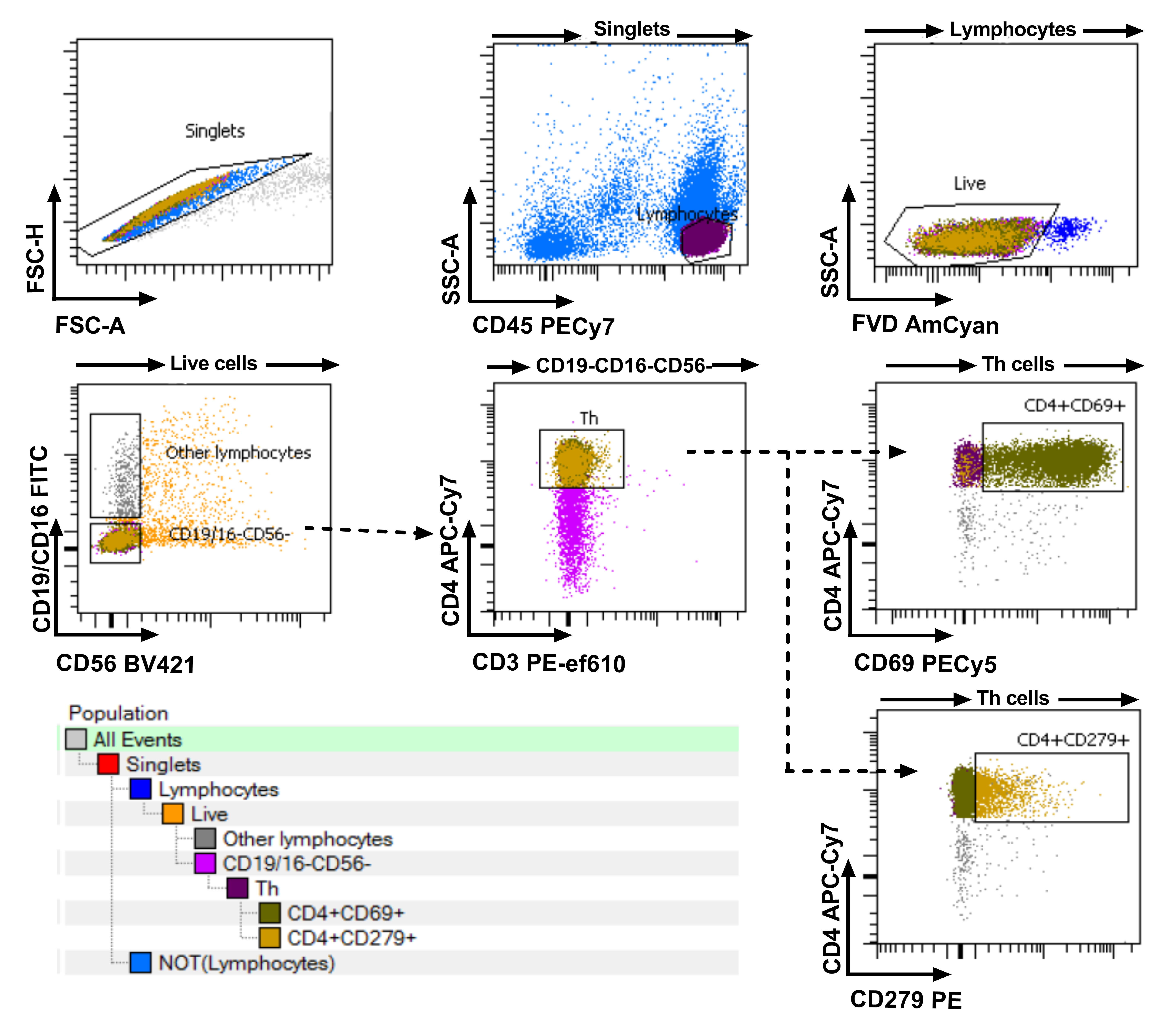

Supplement: Supplementary file 2 — Supplementary information. [file IID3-9-1541-s005.tif]

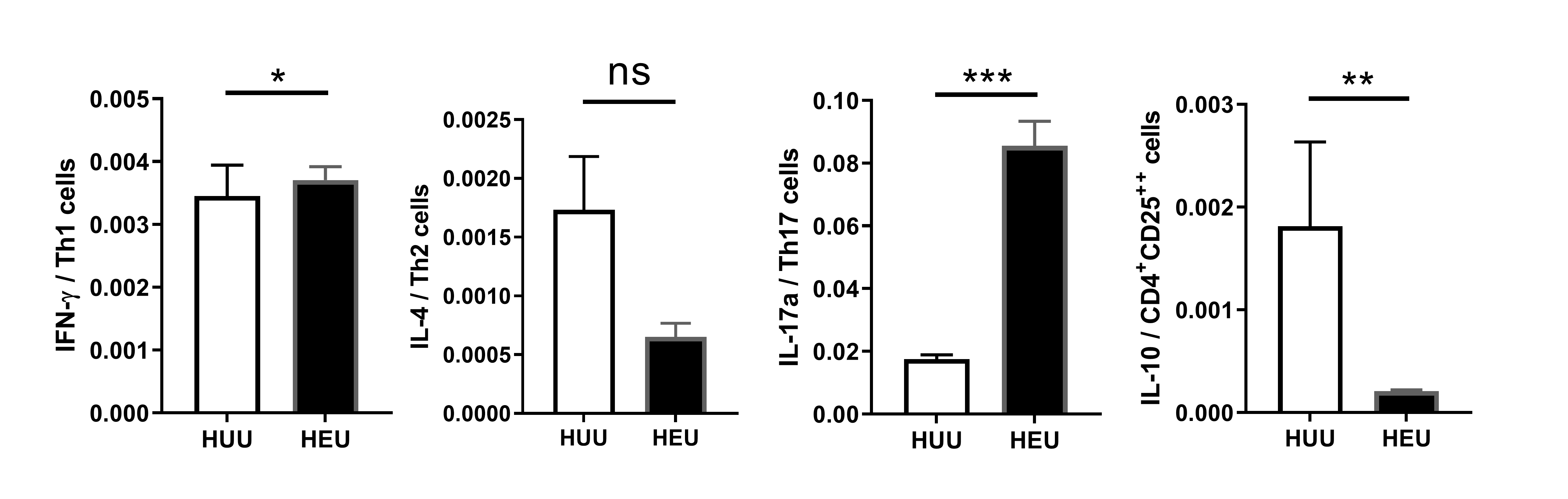

Supplement: Supplementary file 3 — Supplementary information. [file IID3-9-1541-s004.tif]

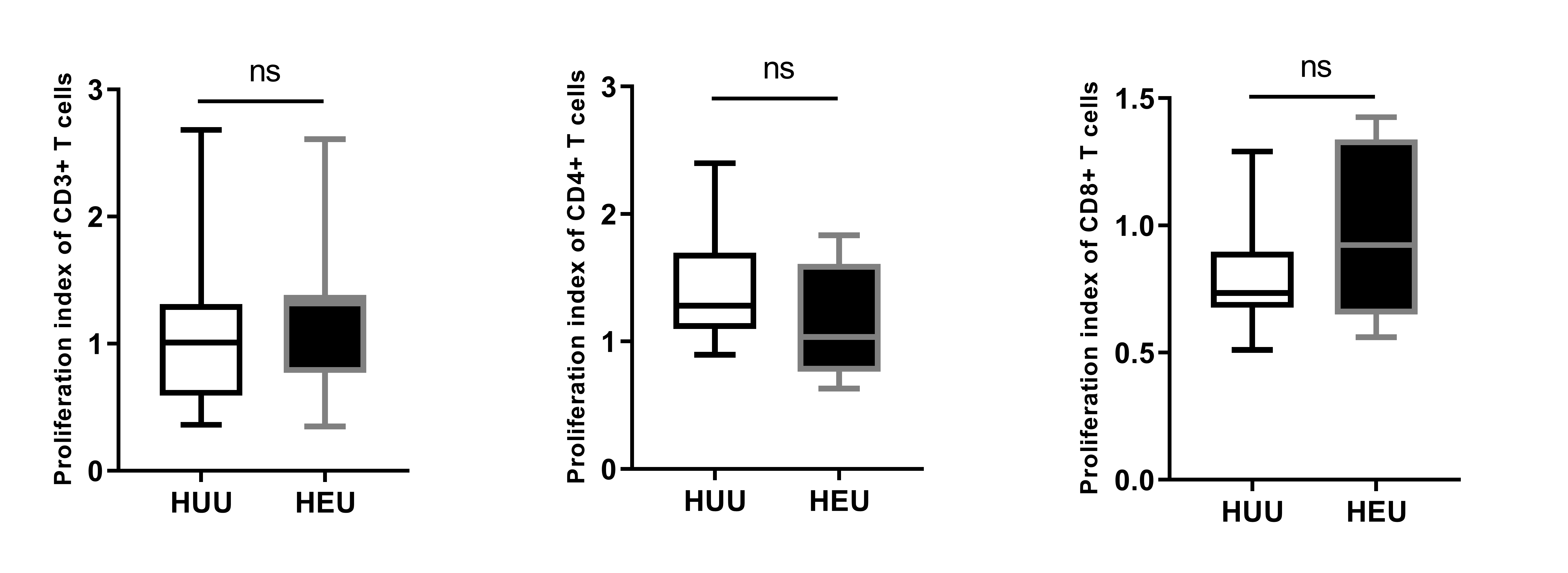

Supplement: Supplementary file 4 — Supplementary information. [file IID3-9-1541-s001.tif]
