# Supplementary material for: Impaired T helper cell responses in human immunodeficiency virus‐exposed uninfected newborns
Source: Immun Inflamm Dis. 2021 Aug 19;9(4):1541–53. doi: 10.1002/iid3.507 (PMC8589403; doi:10.1002/iid3.507)
Supplement: Supplementary file 5 — Supplementary information. [file IID3-9-1541-s002.docx]

**Figure S1. Frequency of Th_0_ cells in HUU and HUE newborns.** a) Representative gating strategy to identify the Th_0_ phenotype from CD4▒^+▒^T cells. b) The percentages of Th_0_ cells (CD45RA▒^+▒^CXCR3^-^CCR4^-^CCR6^-^) among total CD4▒^+▒^T lymphocytes from HEU and HUU newborns are shown. Error bars indicate the median and interquartile range. P-values were calculated using the unpaired Mann–Whitney test. ***P▒<▒0.001.

**Figure S2. Analysis algorithm for the identification of CD69▒^+▒^CD279▒^+▒^activated Th cells.** After stimulation, mononuclear cells were immunostained to identify the percentage of CD69▒^+▒^and CD279▒^+▒^Th cells by flow cytometry. The presented algorithm is a representative image for the experiment. Gray cells (CD19▒^+▒^and CD16▒^+▒^) were used as internal negative controls to discriminate CD69- and CD279- Th cells from positive ones.

**Figure S3.** **Normalized production of cytokines in HUU and HEU newborns.** Relationship between the concentrations of each cytokine and the number of classical corresponding Th cells. P-values were calculated using unpaired Mann–Whitney test to compare groups (HEU vs. HUU newborns). *P▒<▒0.05 and ****P▒<▒0.0001.

**Figure S4**. **Proliferation index in HUU and HEU newborns.** The proliferation index was calculated as the quotient of the percentage of each population in the differentiation Th_1_ medium and unstimulated condition. P-values were calculated using an unpaired Mann–Whitney test to compare groups (HEU vs. HUU newborns). ns, not significant.
